# Supplementary material for: Expansion of cytotoxic tissue-resident CD8+ T cells and CCR6+CD161+ CD4+ T cells in the nasal mucosa following mRNA COVID-19 vaccination
Source: Nat Commun. 2022 Jun 10;13:3357. doi: 10.1038/s41467-022-30913-4 (PMC9186487; doi:10.1038/s41467-022-30913-4)
Supplement: Supplementary file 1 — Supplementary Information [file 41467_2022_30913_MOESM1_ESM.docx]

**Supplementary Table 1.** Participant demographics and health status.

| Variable | Study participants (n= 21) |
| --- | --- |
| Age (median, IQR) | 40 (31-50) |
| Sex, female | 67% (14/21) |
| Body mass index (BMI, median, IQR) | 24.4 (21.5-28.9) |
| Received 2020 fall flu vaccine | 90% (19/21) |
| Diagnosed with COVID-19 in 2020 | 10% (2/21) |
| Self-reported underlying medical conditions | Asthma/allergy (6/21)  Type 2 diabetes (2/21)  Hypertension (2/21)  Autoimmunity (1/21) |
| Dose 2 side effects | None (5/21)  Sore arm (3/21)  Moderate effects including nausea, fever, influenza-like symptoms (13/21) |

**Supplementary Table 2.** Age and Sex-stratified analysis of CD8^+^ Trm and CD4^+^CCR6^+^CD161^+^ cells following SARS–CoV-2 vaccination

| Strata | | Outcome* (V3 vs baseline; beta & 95% CI) | | |
| --- | --- | --- | --- | --- |
|  |  | CD8^+^ CD69^+^CD103^+^ | CD8^+^ CD69^+^CD103^-^ | CD4^+^ CCR6^+^CD161+ |
| Entire cohort | | 0.43 (0.11-0.64) | 0.48 (0.16-0.81) | 0.45 (0.12-0.78) |
| Sex | Males (n=7) | 0.57 (0.08-1.07) | 0.39 (-0.11-0.90) | 0.38 (-0.07-0.83) |
|  | Females (n=14) | 0.39 (0.00-0.78) | 0.51 (0.11-0.90) | 0.49 (0.08-0.90) |
| Age | ≤35 years of age (n=8) | 0.21 (-0.30-0.71) | 0.22 (-0.18-0.62) | 0.42 (-0.14-0.98) |
|  | >35 to <50 years of age (n=6) | 0.85 (0.68-1.02) | 1.00 (0.56-1.44) | 0.55 (0.18-0.91) |
|  | ≥50 years of age (n=7) | 0.45 (-0.06-0.95) | 0.42 (-0.09-0.94) | 0.41 (-0.13-0.95) |

* log10 cells per nasal swab, adjusted as per main analysis

**Supplementary table 3.** List of Flow cytometry antibodies/reagents

| Antibody/reagent | Catalogue No. | Company | Vol/50ul/ Titre |
| --- | --- | --- | --- |
| Anti- Human CD326 (EpCAM) Brilliant Violet 650 (Clone 9C4) | 324226 | Biolegend | 1 |
| Anti-Human CD38 BB515 (Clone HIT2) | 564498 | BD Horizon | 1 |
| Anti- Human Perforin PerCP/Cyanine5.5 (Clone B-D48) | 353314 | Biolegend | 1 |
| Anti- Human CD134 (OX40) PE/Cyanine7 (Clone Ber-ACT35) | 350012 | Biolegend | 1 |
| Anti-Human CD69 PE/Cyanine5 (Clone FN50) | 310908 | Biolegend | 1 |
| Anti-Human CD161 PE (Clone HP-3G10) | 12-1619-42 | eBioscience | 1 |
| Anti-Human Bcl-2 PE (Clone100) | 658708 | Biolegend | 2 |
| Anti-Human CD196 (CCR6) BV421(Clone 11A9) | 562515 | BD Horizon | 1 |
| Anti-Human CD103 (Integrin αE) Brilliant Violet 605 (Clone Ber-ACT8) | 350218 | Biolegend | 1 |
| Anti-Human HLA-DR BV650 (Clone G46-6) | 564231 | BD Horizon | 1 |
| Anti-Human CD154 Brilliant Violet 711(Clone 24-31) | 310838 | Biolegend | 1 |
| Anti-Human CD45 Brilliant Violet 785(Clone HI30) | 304048 | Biolegend | 0.5 |
| Anti-Human CD8a APC/Cyanine7 (Clone RPA-T8) | 301016 | Biolegend | 0.5 |
| Anti-Human CD3 Alexa Fluor® 700 (Clone UCHT1) | 300424 | Biolegend | 0.5 |
| Anti-Human Ki-67 Alexa Fluor® 647 (Clone B56) | 561126 | BD Pharmingen | 2 |
| Anti-Human CXCR5 (CD185)  BUV395(Clone RF8B2) | 740266 | BD OptiBuild | 1 |
| Anti-Human CD4 BUV496 (Clone SK3) | 612936 | BD Horizon | 0.5 |
| Anti-Human CD279 (PD-1) BUV737 (Clone EH12.1) | 612791 | BD Horizon | 1 |
| LIVE/DEAD™ Fixable Aqua Dead Cell Stain Kit | L34957 | Invitrogen | 1:500 |
| Antigen-specific staining antibodies | | | |
| Anti-Human TNF-α FITC (Clone (MAb11) | 11-7349-41 | eBioscience | 1 |
| Anti-Human Perforin PerCP/Cyanine5.5 (Clone B-D48) | 353314 | Biolegend | 1 |
| Anti-Human IFN-γ PE/Cyanine7(Clone B27) | 557643 | BD Pharmingen | 1 |
| Anti-Human CD69 PE/Cyanine5 (Clone FN50) | 310908 | Biolegend | 1 |
| Anti-Human CD107a PE-CF594 (Clone H4A3) | 562628 | BD Horizon | 2 |
| Anti-Human IL-22 PE (Clone 22URTI) | 12-7229-42 | eBioscience | 1 |
| Anti-Human IL-17A BV421(Clone ) | N49-653 | BD Pharmingen | 1 |
| Anti-Human CD103 (Integrin αE) Brilliant Violet 605 (Clone Ber-ACT8) | 350218 | Biolegend | 1 |
| Anti-Human CD8 BV650 (Clone RPA-T8) | 564231 | BD Horizon | 0.5 |
| Anti-Human CD154 (CD40L) Brilliant Violet 711(Clone 24-31) | 310838 | Biolegend | 1 |
| Anti-Human CD45 Brilliant Violet 785(Clone HI30) | 304048 | Biolegend | 0.5 |
| Anti-Human CD3 APC-H7 (Clone: SK7) | 560176 | BD Pharmingen | 0.5 |
| Anti-Human IL-2 APC-R700 (Clone MQ1-17H12) | 565136 | BD Horizon | 1 |
| Anti-Human Ki-67 Alexa Fluor® 647 (Clone B56) | 561126 | BD Pharmingen | 2 |
| Anti-Human CD45RO  BUV395(Clone UCHL1) | 564291 | BD Horizon | 1 |
| Anti-Human CD4 BUV496 (Clone SK3) | 612936 | BD Horizon | 0.5 |
| LIVE/DEAD™ Fixable Aqua Dead Cell Stain Kit | L34957 | Invitrogen | 1/500 |


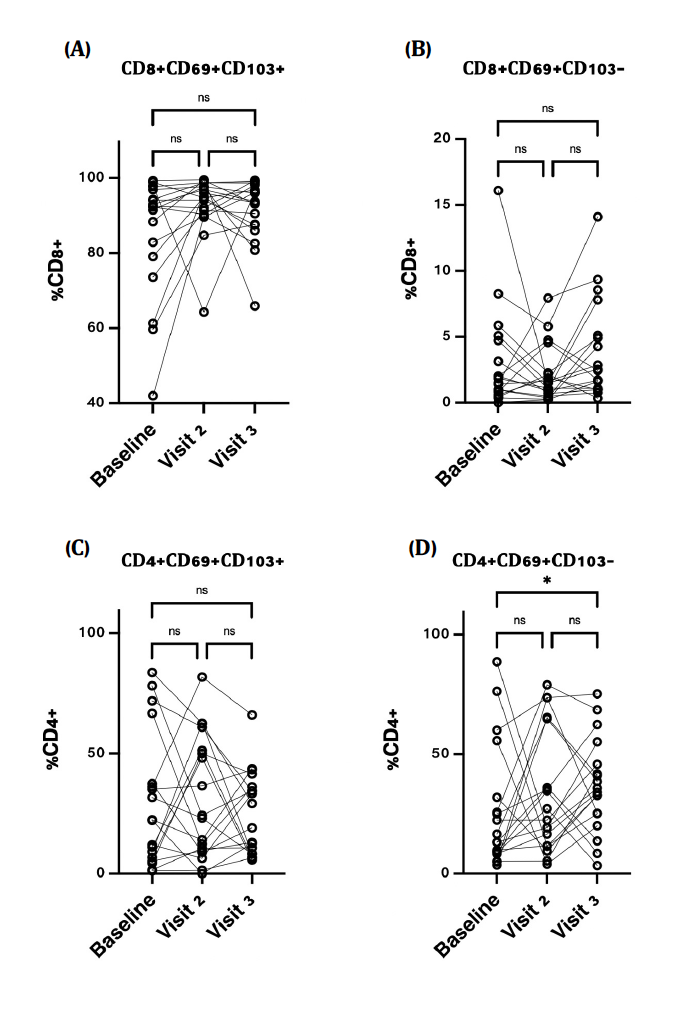


**Supplementary Figure 1.** Frequencies of nasal CD4 and CD8 Trm T cells post SARS-COV-2 vaccination (n=21). No significant differences were observed in the proportions of (A) CD8^+^CD69^+^CD103^+^, (B) CD8^+^CD69^+^CD103^-^, (C) CD4^+^CD69^+^CD103^+^ Trm at baseline, visit 1 and 2 while a significant difference was observed for (D) CD4^+^CD69^+^CD103^+^ Trm T cells at baseline and visit 2 (* P>0.01) . Tukey's multiple comparisons test was used (ns: non-significant).


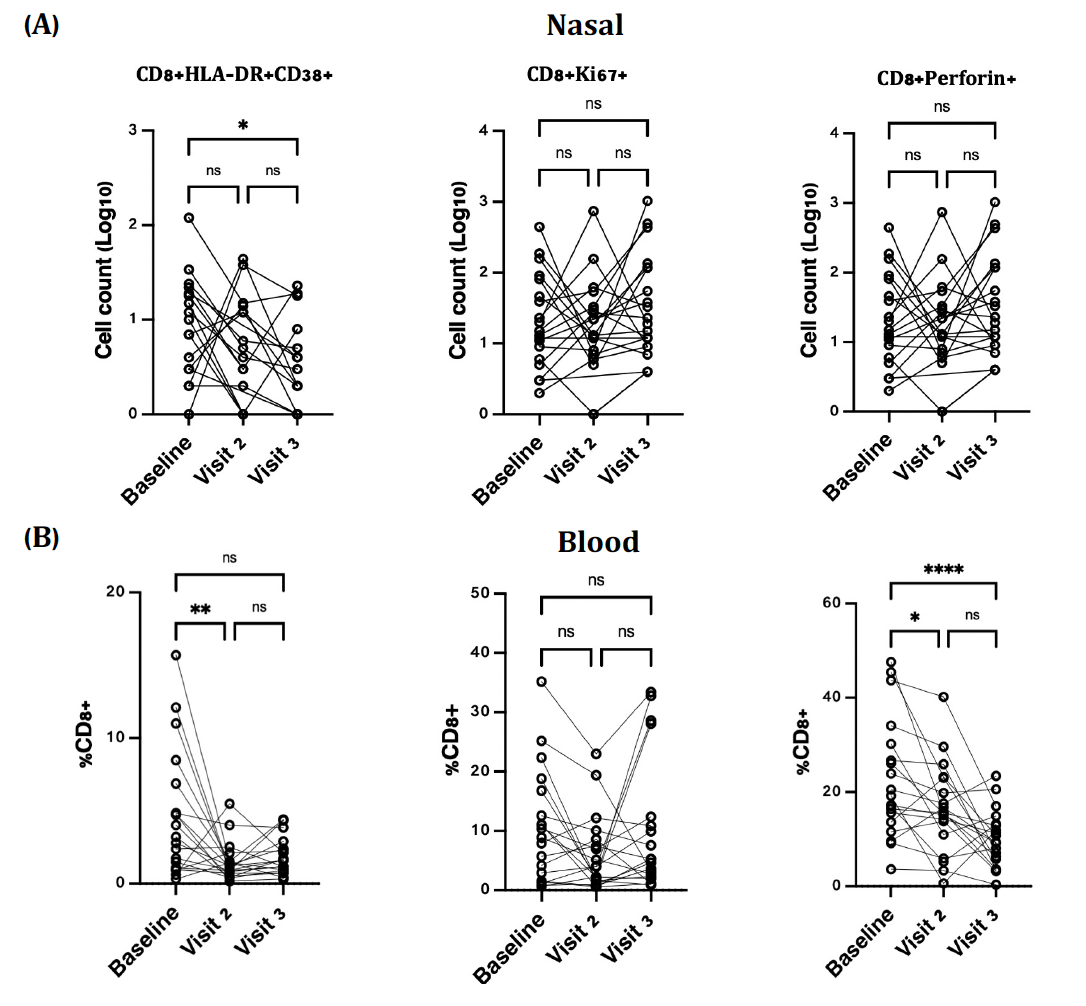


**Supplementary Figure 2.** CD8^+^ T cell activation defined by expression of HLA-DR and CD38, proliferation, based on the expression of Ki-67 and cytotoxicity, defined by the expression of perforin in cells isolated from (A) nasopharyngeal swabs and (B) peripheral blood amongst vaccinated individuals (n=21) following SARS-COV-2 vaccination. Significant increases in nasal CD8^+^ HLA-DR^+^CD38^+^ T cell abundance was observed at baseline and visit 3 (* p<0.01) (A), while in the blood, a significant increase in proportion of CD8^+^ HLA-DR^+^CD38^+^ T cells was observed at baseline and visit 2 (**p<0.001) (B) Furthermore, a significant increase in the proportion of peripheral blood cytotoxic CD8^+^Perforin^+^ T cells was observed at baseline and visit 1 as well as baseline and visit 3 (* p<0.01, ****p<0.0001) Tukey's multiple comparisons test was used (ns: non-significant).


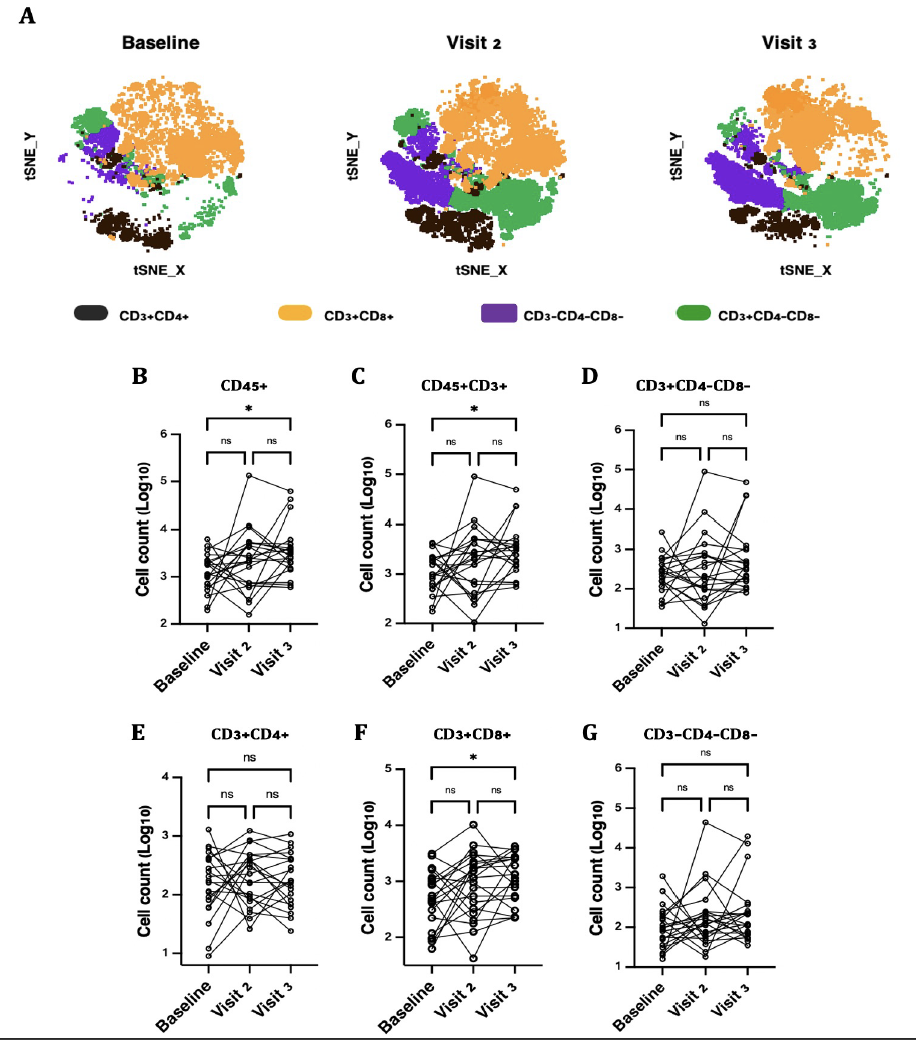


**Supplementary Figure 3.** Nasal immune cell clustering and cell counts per swab following SARS-COV-2 vaccination. (A) t-distributed stochastic neighbour embedding (tSNE) plots of integrated nasal CD45+ immune cells showing shifts in CD3^+^CD4^+^, CD3^+^CD8^+^, CD3^-^CD4^-^CD8^-^ and CD3^+^CD4^-^CD8^-^ immune cell clusters from vaccinated individuals (n=21) at baseline, visit and visit 3. (B) CD45+ (* P>0.01) (C) CD45^+^CD3^+^ (* P>0.01) and (F) CD3^+^CD8^+^ (* P>0.01) cells were significantly increased following the 2^nd^ vaccination dose while no changes were observed for (D) CD3^+^CD4^-^CD8^-^, (E) CD3^+^CD4^+^ and (G) CD3^-^CD4^-^CD8^-^ immune cell subsets (ns: non-significant).


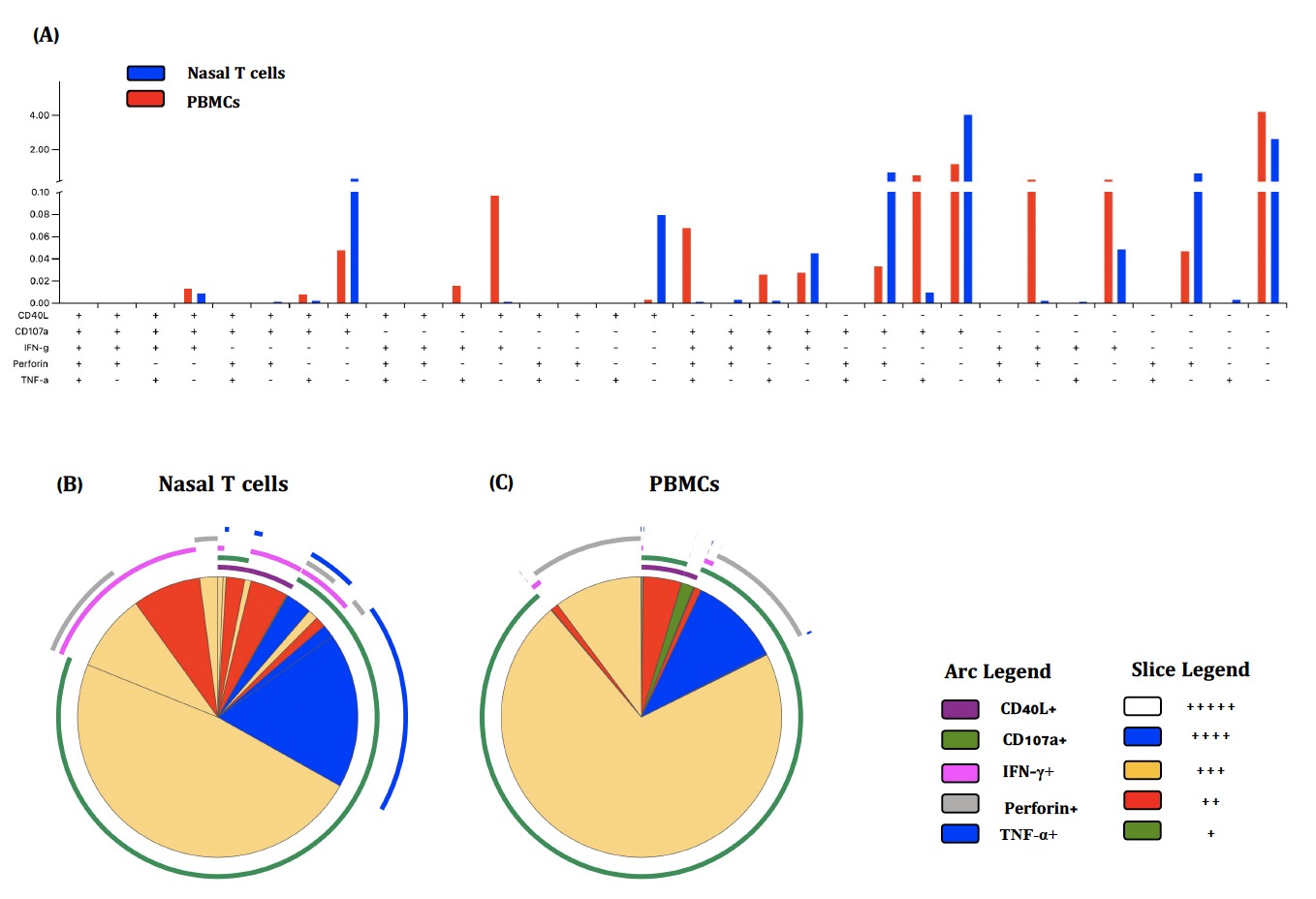


**Supplementary Figure 4.** SARS-CoV-2 spike-specific CD8^+^ T cell responses ~6 months post-vaccination. Represented on bar graph (A) is the polyfunctional profiling of the mean frequencies of up to 31 phenotypic combinations of nasal and systemic antigen-specific CD8^+^ T cells. The arcs on the pie charts indicate the proportion of (B) nasal and (C) systemic T cells expressing CD40L, CD107a


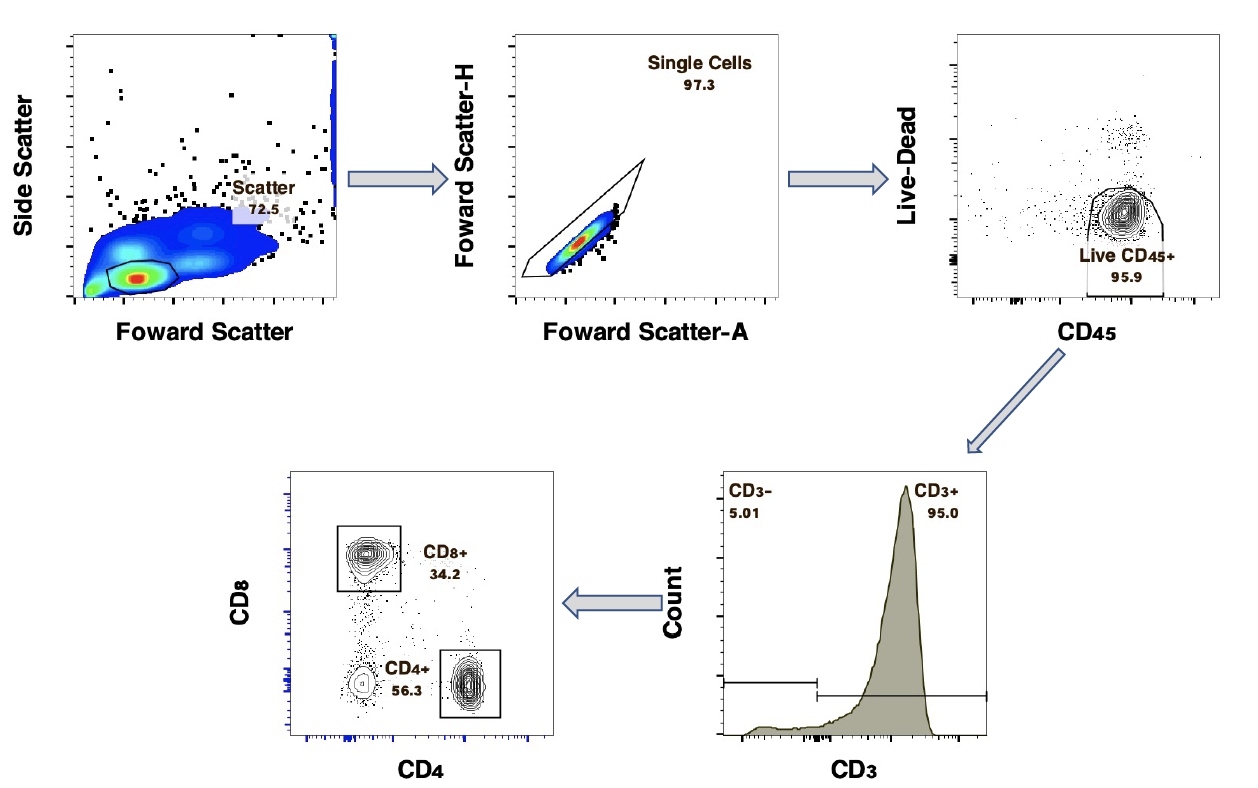


**Supplementary Figure 5.** Representative gating strategy of peripheral blood mononuclear cells showing the exclusion of non-conventional T cells.
